# Nasal high flow therapy use in wards in patients with chronic obstructive pulmonary disease may spare ICU resources

**DOI:** 10.1111/crj.13458

**Published:** 2021-11-15

**Authors:** Matthew W. Trump, Iaswarya Ganapathiraju, Julie A. Jackson, Kate Branick, Matt Taylor, Trevor W. Oetting, Carol A. Pelaez

**Affiliations:** ^1^ Pulmonary and Critical Care Medicine The Iowa Clinic Des Moines IA USA; ^2^ Pulmonary and Critical Care Medicine UnityPoint Health Des Moines IA USA; ^3^ University of Iowa – Des Moines Internal Medicine Program UnityPoint Health Des Moines IA USA; ^4^ Respiratory Therapy UnityPoint Health Des Moines IA USA; ^5^ Trauma Surgery The Iowa Clinic Des Moines IA USA; ^6^ Trauma Services UnityPoint Health Des Moines IA USA

**Keywords:** acute hypoxic respiratory failure, chronic obstructive pulmonary disease, ICU, nasal high flow

## Abstract

Nasal high flow therapy has been previously studied for the management of acute hypoxic respiratory failure in patients with chronic obstructive pulmonary disease but the data regarding its use outside of the intensive care unit are sparse. We aimed to evaluate safety and efficacy of nasal high flow therapy outside of the intensive care unit in patients with acute hypoxic respiratory failure and known chronic obstructive pulmonary disease. We conducted a retrospective matched historic cohort study of adult patients with diagnosed chronic obstructive pulmonary disease presenting with acute hypoxic respiratory failure between December 2017 to June 2019, after the initiation of a new protocol, which allowed patients to be managed with nasal high flow therapy on the medical/surgical wards instead of transferring them to the ICU per prior standard of care. Nasal high flow therapy was initiated either in the emergency department or on the medical/surgical wards. Patients were matched with historical cohorts who were managed with prior standard of care based on age, body mass index, comorbidities, and home oxygen use. Primary outcome of interest was difference in rates of mechanical ventilation. Secondary outcomes included hospital length of stay, total number of days spent in the intensive care unit, and in‐hospital mortality. A total of 90 patients met study inclusion criteria and were matched to 90 historical control patients. Among the study group, 8% required mechanical ventilation versus 9% in the control group (*p* = 0.79). Hospital length of stay was 7 days in study group versus 6 days in control group (*p* = 0.02), and in‐hospital mortality was the same in both study and control groups at 12% (*p* = 0.99). Nineteen percent of study group patients required ICU level of care at any time during the admission compared with 49% of control group (*p* < 0.001). Nasal high flow therapy use in patients with acute hypoxic respiratory failure and underlying chronic obstructive pulmonary disease outside of the intensive care unit may spare ICU resources and cost without delay in definitive care such as mechanical ventilation.

## 1 INTRODUCTION

Chronic obstructive pulmonary disease (COPD) is a leading cause of morbidity and mortality in the United States, accounting for approximately 150 000 deaths per year.^1^ In 2020, the healthcare cost attributable to COPD was estimated to be approximately $50 billion.^2^ In addition, acute exacerbations of COPD (AECOPD) are associated with increased risk of myocardial infarction, arrhythmia, stroke, and sudden cardiac death, further adding to the clinical and economic burden of COPD.^3^, ^4^ The inflammation that occurs during AECOPD leads to increased mucus production and bronchoconstriction, ultimately causing respiratory compromise including acute hypoxic respiratory failure (AHRF) and acute hypercapnic respiratory failure.^5^

AHRF in COPD patients can result from AECOPD or other causes such as acute heart failure exacerbations and infections. It is often managed with supplemental oxygen therapy via low‐flow nasal cannulas or face masks, or, in moderate to severe cases, with non‐invasive ventilation (NIV) systems such as bilevel positive airway pressure (BiPAP). These treatments are complicated by the fact that hyperoxygenating patients with COPD can lead to respiratory depression and worsening hypercapnia.^6^, ^7^ A variety of mechanisms for this deleterious effect of oxygen therapy have been proposed, but there is not clear consensus as to the true pathophysiology behind this response. Multiple studies have demonstrated, however, the importance of titratable oxygen delivery with close monitoring of oxygen saturations (SpO_2_) for COPD patients.^8^

Over the last decade, there has been increased interest in the use of nasal high flow (NHF) oxygen therapy in management of AHRF. NHF is a noninvasive respiratory support device that can deliver up to 60 L/min of heated humidified oxygen via nasal prongs with a titratable fraction of inspired oxygen (FiO_2_) between 21% and 100%.^9^ Therapy with NHF has multiple benefits, including decreased work of breathing, improved lung mechanics, and reduced dead space.^10^, ^11^ NHF has also been associated with lower intubation rates and decreased mortality in management of AHRF in patients with and without COPD.^10^, ^11^

With its growing popularity, NHF has been applied to the management of AHRF in a variety of clinical situations, including post‐cardiac surgery, post‐extubation, and acute congestive heart failure exacerbations.^9^ There is also limited but promising data regarding use of NHF in AECOPD. In a retrospective study comparing acidotic and non‐acidotic hypercapnic patients with AECOPD who failed NIV, Braunlich et al. found that a trial of NHF was associated with improved pH and PaCO_2_.^12^ In a small randomized study of patients with AECOPD, Yang et al. found that patients who received NHF had improvements in diaphragmatic fatigue when compared with patients with conventional oxygen therapy.^13^ Additionally, in a prospective study of 10 patients with AECOPD, Pandya et al. found that NHF was well tolerated by patients.^14^

Despite increasing evidence regarding the safety and efficacy of NHF in COPD patients, use of NHF has been largely limited to intensive care units (ICU) and is less commonly applied for patients with AHRF outside of the ICU. The present study evaluates the role of NHF for patients with COPD and AHRF, with the hypothesis that use of NHF outside of the ICU would result in lower intubation rates and would reduce the burden of care in the ICU.

## 2 METHODS

### 2.1 Study design

We conducted a retrospective, matched historic control study at a Level I trauma center in the Midwest, USA. The study facility is a tertiary hospital with 370 total hospital beds, a 33‐bed emergency department (ED), and a 36‐bed intensive care unit (ICU). Staffing ratios at our hospital are approximately one registered nurse (RN) per two patients in the ICU (1:2) and 1 RN per 5 (1:5) on the medical/surgical wards. Patients were included if they were 18 years of age or older with a known diagnosis of COPD and presented to the hospital with acute hypoxic respiratory failure. Exclusion criteria were patients without prior history of COPD, admitted to the ICU from the ED, tracheostomy‐dependent, treated with NHF therapy for <2 h or treated in the ED only, and treated with NHF for end‐of‐life cares only. Study patients were then matched with historical controls based on age, body mass index, comorbidities including COPD, and home oxygen therapy. The primary outcome of interest was difference in rate of invasive mechanical ventilation between study and comparison controls. Other outcomes of interest included hospital length of stay, total number of days spent in the ICU, and in‐hospital mortality. The study protocol was approved by institutional review board at the study hospital and the informed consent process was waived given the retrospective nature of the study.

### 2.2 Study intervention

Study patients were treated with NHF therapy outside the ICU, which included treatment in the ED and on the medical/surgical wards during the study period (December 2017–June 2019). The comparison group included historical control patients treated per standard care prior to initiation of NHF outside the ICU (2015–2017); this included patients who received low‐ or moderate‐flow nasal cannula up to 15 L/min on the medical/surgical wards or patients who received NHF therapy in the ICU. During the study phase, NHF therapy was delivered via the AIRVO2 system (Fisher & Paykel, Auckland, New Zealand). This device can deliver up to 60 L/min of flow and FiO_2_ of up to 100%.

The use of NHF was implemented in the ED and medical/surgical wards of our hospital prior to the initiation of this study, beginning in December 2017 and rolling out to all units by summer of 2018 per the protocol described by Jackson et al.^15^ (see Supporting Information). Our protocol permits management of patients on nasal high flow on the medical/surgical wards with arterial pH > 7.3. Therapy with NHF was initiated in the ED or on the medical/surgical wards per protocol when patients required >4 L/min of oxygen flow or >36% FiO_2_. Patients were started on NHF with flow rate of 50 L/min and FiO_2_ of at least 10% higher than previous device, then titrated as needed. Per protocol, all patients placed on NHF therapy required evaluation by a respiratory therapist every 4 h while on the therapy. This evaluation included patient vitals, NHF device settings, modified Borg dyspnea scale score, and patient comfort level with device. An established team of study personnel evaluated safety and adverse events of NHF therapy regularly to monitor and ensure real‐time response to safety concerns.

### 2.3 Study variables

Demographic variables were collected for all patients and included age, gender, and BMI. We also tracked clinical factors including admitting specialty and initial APACHE score at the time of NHF initiation, calculated using appropriate data points obtained from the record. Patients were asked about home oxygen therapy by respiratory therapy at time of NHF initiation and usage was tracked. Comorbid conditions were obtained from admission note or from problem list at time of admission; these included congestive heart failure with preserved or reduced ejection fraction, past and present history of cancer of any kind (lung, breast, skin, etc.), sleep apnea diagnosed with prior sleep study, and current smoking status. Initial vitals including heart rate, respiratory rate, and oxygen saturation (SpO_2_) as obtained at time of presentation to the emergency department were collected through retrospective chart review, as were initial arterial partial pressure of carbon dioxide (pCO_2_), serum bicarbonate (HCO_3_) levels, and arterial pH. “Any ICU days” were considered a duration >24 h spent in the unit.

### 2.4 Statistical analysis

A priori power analysis was conducted prior to initiation of study and indicated a sample size of 84–105 patients was required to detect a difference in rates of invasive ventilation for a moderate effect size with power of 0.80 based on previous studies.^16^ All analyses were performed with IBM SPSS Basic Statistics for Windows, version 20.0 (IBM Armonk, New York). Descriptive statistics were examined and reported for continuous data as medians and interquartile ranges (IQR); categorical data were reported as counts and percentages. Statistical tests were two‐tailed and based on a 0.05 significance level. Because data were not normally distributed and sample sizes were unequal, differences between medians were assessed using the Kruskal–Wallis one‐way analysis of variance. Differences between nominal variables were assessed using the chi‐square testing. Correlations were assessed using Pearson coefficient (*r*). Mean pH values were normally distributed, and Student's *t* test was used to assess for statistical significance.

## 3 RESULTS

### 3.1 Demographics and clinical characteristics

**FIGURE 1:**
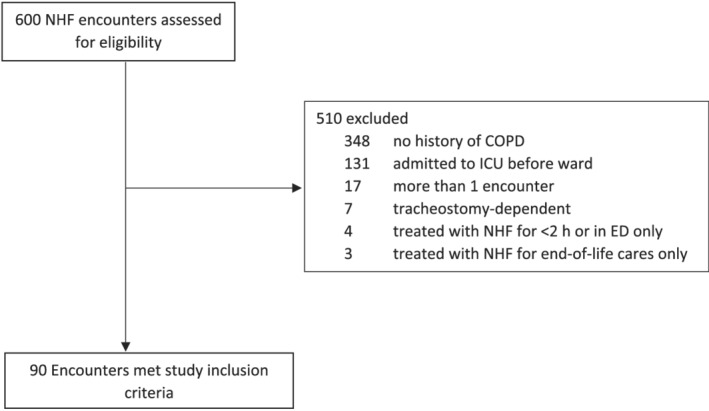
Consort diagram

During the study period, a total of 600 unique patient encounters were screened for inclusion in the study control; 90 patients met study inclusion criteria. As shown in Figure 1, the majority of excluded patients had no known history of COPD or were admitted to the ICU from the ED. The median age of the study group was 71 years [IQR 63–79] and 48% of patients were male. Study patients were compared with a historical control (*N* = 90) with similar demographics (median age of 71 years [IQR 64–79] and 47% male). Baseline characteristic comparison between the study and control groups is shown in Table 1. There were no statistically significant differences between the two groups with regard to age, gender, BMI, home oxygen use, or comorbidities. When compared with the control group, the treatment group had lower initial oxygen saturation as measured in the ED (91% versus 93%, *p* < 0.001) and lower levels of partial pressure of carbon dioxide (PCO_2_) as measured on initial arterial blood gas analysis (50 mmHg versus 44 mmHg, *p* = 0.002). The control group had a mean pH of 7.345 versus 7.403 in the study group (*p* < 0.001), and median pH values for the control group were 7.36 (IQR 7.27–7.45) versus 7.41 (IQR 7.33–7.49) in the study group.

**TABLE 1:** Characteristics of control and study groups at baseline

	Control sample (*n* = 90)	Study sample (*n* = 90)	*p* value
Male, *n* (%)	42 (47%)	43 (48%)	0.88
Age in years, median (IQR)	71 (64–79)	71 (63–79)	0.92
Body mass index (BMI), median (IQR)	29 (25, 36)	28 (23–36)	0.38
Initial APACHE score, median (IQR)	–	6.9 (5.1–8.9)	–
Admitting service, *n* (%)			0.31
Pulmonology	46 (51%)	39 (43%)	
Internal medicine	40 (44%)	46 (51%)	
Surgical service	4 (5%)	5 (6%)	
Home oxygen therapy, *n* (%)	51 (57%)	46 (51%)	0.46
ED initial heart rate, median (IQR)	93 (83–113)	101 (83–111)	0.53
ED initial respiratory rate, median (IQR)	23 (20–28)	22 (18–24)	0.30
ED initial SpO_2_, median (IQR)	93 (89–96)	91 (84–92)	<0.001
Initial pH, mean	7.345	7.403	<0.001
Initial pH, median (IQR)	7.36 (7.27–7.45)	7.41 (7.33–7.49)	
Initial PCO_2_, median (IQR)	50 (43–64)	44 (36–53)	0.002
Initial HCO_3_, median (IQR)	27 (24–32)	28 (24–32)	0.59
Do‐not‐resuscitate (DNR) prior to or during stay, *n* (%)	35 (39%)	39 (43%)	0.55
Comorbidities, *n* (%)			
Congestive heart failure (CHF)	40 (44%)	38 (42%)	0.77
Cancer (any)	22 (24%)	29 (32%)	0.25
Obstructive sleep apnea (OSA)	29 (32%)	25 (28%)	0.52
Current smoker	30 (33%)	31 (34%)	0.88

Abbreviations: HCO_3_, hydrogen bicarbonate; PCO_2_, partial pressure of carbon dioxide; SpO_2_, oxygen saturation.

In the study group, therapy with NHF was initiated approximately 4.7 h after arrival to ED and median duration of therapy was 49 h. Therapy was initiated in the ED for 44 patients (49%) and on the medical/surgical wards for 46 (51%) patients.

### 3.2 Outcomes

Primary study outcomes are highlighted in Table 2. There was no statistically significant difference between need for mechanical ventilation (8% vs. 9%, *p* = 0.79) or in‐hospital mortality (12% vs. 12%, *p* = 0.99) between the study group and the control group. Only 19% of patients in the study group required ICU‐level care at any time during the admission, compared with 49% of control group (*p* < 0.001). The rate of transfers to the ICU from the ward after initial admission was similar between the study group and the control group (19% vs. 14%, *p* = 0.43).

**TABLE 2:** Key outcome comparison between control and study groups

	Control sample (*n* = 90)	Study sample (*n* = 90)	*p* value
Hospital days, median (IQR)	6 (4, 8)	7 (5, 13)	0.02
Admission to ICU before ward, *n* (%)	32 (36%)	0 (0%)	<0.001
Admission to ICU after ward, *n* (%)	13 (14%)	17 (19%)	0.43
Any ICU days, *n* (%)	44 (49%)	17 (19%)	<0.001
Mortality, *n* (%)	11 (12%)	11 (12%)	0.99
Hours between arrival and need for >4 L oxygen support, median (IQR)	2.7 (0.1, 27.8)	0.7 (0.1, 31.5)	0.57
Intubated during hospitalization, *n* (%)	8 (9%)	7 (8%)	0.79
Initiation of NHF, *n* (%)			–
Emergency department (ED)	–	44 (49%)	
Medical/surgical ward	–	46 (51%)	
Hours between arrival and NHF initiation, median (IQR)	–	4.7 (1.9, 46.2)	–
Hours of NHF therapy, median (IQR)	–	49 (22, 101)	–
Initial Visual Analog Scale score on NHF therapy, median (IQR)	–	3 (1, 4)	–

## 4 DISCUSSION

Nasal high flow therapy has been shown to be beneficial in the management of acute hypoxic respiratory failure in various care settings. Previous standard of care was to limit use of NHF therapy to the ICU setting only as its safety and efficacy had not yet been studied in detail outside of the ICU. In the present study, the use of NHF therapy outside the ICU allowed a significant number of study patients to be admitted to the ward and remain on the ward through their hospitalization. Patients in the study group did not differ statistically from the control group in terms of escalation to mechanical ventilation or in‐hospital mortality, suggesting that the use of NHF therapy outside of the ICU was safe, efficacious, and reduced the need for ICU‐level care and resources.

To our knowledge, this is the first study evaluating the use of NHF therapy for management of AHRF in COPD patients on the medical/surgical wards. Use of NHF in patients with COPD has been previously studied in the ICU setting and studies have demonstrated that NHF therapy does not increase risk of intubation or delay definitive therapy compared with NIV, regardless of comorbidities or severity of COPD.^17^, ^18^, ^19^ Additionally, other studies have evaluated NHF therapy in the outpatient setting for patients with COPD and chronic hypercapnia and they found no increased risk of adverse outcomes in those treated with NHF therapy compared with NIV therapy.^20^, ^21^

Despite previous findings, there is still uncertainty and sparse published data regarding the safety of NHF therapy outside of the ICU. The results of our present study suggest that NHF therapy can be safely delivered in the medical/surgical wards for patients with underlying COPD who may be especially sensitive to the complications that arise from hyperoxygenation. This is particularly efficacious when delivered in the context of a regimented protocol driven by respiratory therapists and in collaboration with pulmonologists.^15^

There are several limitations to our study. First, this was a retrospective study and not a randomized controlled trial, thus the sample may be prone to selection bias. Second, the criteria for transfer to ICU was not specified by the therapy protocol and the decision to transfer was left to the discretion of the attending physician. Given the multifactorial nature of ICU transfers as well as potential lower threshold for transfer to the ICU given that NHF therapy on the wards was a relatively new concept, results from this study must be viewed with caution. However, this potentially further validates study results showing lower ICU utilization in the study group. The control group did have lower baseline pH and higher baseline PCO_2_ than the study group; mean pH of 7.345 in the control group versus 7.403 in the study group, and median pH of 7.36 in the control group and 7.41 in the study group. These differences are unlikely to be clinically significant, however could have contributed to part of the differences in rates of ICU transfer between the two groups. Finally, this study focused on patients with underlying COPD presenting with AHRF due to any cause, not exclusive to acute COPD exacerbations as the primary diagnosis. Caution must therefore be utilized before generalizing these findings to patients in acute exacerbation, particularly those with moderate to severe respiratory acidosis as these patients were excluded from this study. Future studies in this area should address AHRF in patients with AECOPD specifically. It is also important to note that at the time of this study being conducted, a regimented protocol for weaning patients from NHF therapy had not been implemented yet at the study facility. A standardized weaning protocol has the potential to decrease length of stay and should be studied further in this context.

In conclusion, nasal high flow is an important therapy for the management of acute hypoxic respiratory failure in patients with underlying chronic obstructive pulmonary disease. This therapy can be initiated and administered outside the ICU setting, when delivered in the context of a comprehensive protocol with appropriate indications for therapy and monitoring. The standardization and expansion of NHF therapy to medical/surgical wards may spare ICU resources and reduce ICU‐associated cost without delaying definitive care.

## CONFLICT OF INTEREST

Ms. Jackson and Dr. Trump disclose that they have served as consultants and advisory board members for Fisher & Paykel HealthCare.

## ETHICS STATEMENT

This study involving human participants was in accordance with the ethical standards of our institutional review board and/or national research committee and with the 1964 Helsinki declaration and its later amendments or comparable ethical standards. Ethical approval for the study was obtained from our institutional review board. For this type of study formal informed consent is not required to participate nor publish.

## Supporting information


**Data S1.** Supporting Information

## Data Availability

The data that support the findings of this study are available from the corresponding author upon reasonable request.
