# Supplementary material for: Nasal high flow therapy use in wards in patients with chronic obstructive pulmonary disease may spare ICU resources
Source: Clin Respir J. 2021 Nov 15;16(2):116–22. doi: 10.1111/crj.13458 (PMC9060061; doi:10.1111/crj.13458)
Supplement: Supplementary file 1 — Data S1. Supporting Information [file CRJ-16-116-s001.docx]

ONLINE SUPPLEMENT I

Indications/Contraindications for nasal high flow therapy:

Patient populations that will potentially benefit from nasal high flow therapy include:

- Patients where traditional nasal cannula does not meet patient flow demand and/or FiO2 requirement.
- Tracheostomy patients, who may require higher flows and/or heated, humidified gas.
- COPD, asthmatics, and a subset of patients that have viral diseases (i.e. Influenza, COVID-19, etc.) that have normal lung compliance but have severe symptomatic hypoxemia and other patients with cardiac and pulmonary diseases as deemed appropriate by Pulmonary Physicians.
- Patients with 3 or more rib fractures or pulmonary contusions that need further splinting or stenting of the airways during healing process. This must be documented by X-ray report, CT report or trauma physician notes in the ICU or on the floor.
- Patients who require hydration of thickened secretions.
- Dyspnea and/or increased work of breathing.
- Hypoxemia requiring >4 lpm oxygen.
- P/F ratio >150.
- Intolerance to noninvasive mechanical ventilation or in the case of emergency department patients, anyone that comes in via EMS with CPAP +7 or less.
- Palliation for air hunger, dyspnea, and/or hypoxia at the end of life. Patients that are DNR/DNI and in whom there is treatment in place and the patients need this support to see if therapy will work (i.e. on anti-viral(s) and need a couple of days to see if medication will work).

Patient populations that may be inappropriate include:

- Patients who are obtunded and unable to maintain their airway.
- Patients with severe respiratory acidosis (ventilatory failure).
- Patients with suspected facial fractures or skull fractures.
- Patients in shock.
- Patients with upper airway obstruction.

Procedure

- The therapy can be used outside the Intensive Care setting. It will require a consult for management of the therapy by Adult Pulmonary/Critical Care or Trauma Services providers. Patient placement for therapy will always be at the discretion of the adult pulmonary/critical care or trauma services providers and will be documented in the EMR.

Initial settings:

- Flow – should start at 50Lpm and adjust for patient effect. The RT may increase the flow if patient continues with accessory muscle use and increased work of breathing.
- FiO2 – set the FiO2 at least 10% higher than the previous device that the patient was on prior to initiating nasal high flow. Up to 100% FiO2 can be used to start with if needed to achieve SpO2 goal.
- SpO2 goal – will depend on patient condition and will need to be discussed with providers.
- Temperature –All device heaters should be allowed to warm to at least 33°C before placing on patient.  Start at 37°C and maintain for duration of therapy. If patient complains, may adjust down to 34°C or even down to 31°C if necessary per patient comfort.
